# Melatonin suppression and sleepiness in children exposed to blue‐enriched white LED lighting at night

**DOI:** 10.14814/phy2.13942

**Published:** 2018-12-16

**Authors:** Sang‐il Lee, Kouhei Matsumori, Kana Nishimura, Yuki Nishimura, Yuki Ikeda, Taisuke Eto, Shigekazu Higuchi

**Affiliations:** ^1^ Department of Human Science, Faculty of Design Kyushu University Fukuoka Japan; ^2^ Laboratory of Environmental Ergonomics Faculty of Engineering Hokkaido University Sapporo Japan; ^3^ Graduate School of Integrated Frontier Sciences Kyushu University Fukuoka Japan; ^4^ Research Fellow of the Japan Society for the Promotion of Science Fukuoka Japan

**Keywords:** Child, circadian rhythm, color temperature, light, sleep

## Abstract

Light‐induced melatonin suppression in children is reported to be more sensitive to white light at night than that in adults; however, it is unclear whether it depends on spectral distribution of lighting. In this study, we investigated the effects of different color temperatures of LED lighting on children's melatonin secretion during the night. Twenty‐two healthy children (8.9 ± 2.2 years old) and 20 adults (41.7 ± 4.4 years old) participated in this study. A between‐subjects design with four combinations, including two age groups (adults and children) and the two color temperature conditions (3000 K and 6200 K), was used. The experiment was conducted for two consecutive nights. On the first night, saliva samples were collected every hour under a dim light condition (<30 lx). On the second night, the participants were exposed to either color temperature condition. Melatonin suppression in children was greater than that in adults at both 3000 K and 6200 K condition. The 6200 K condition resulted in greater melatonin suppression than did the 3000 K condition in children (*P *<* *0.05) but not in adults. Subjective sleepiness in children exposed to 6200 K light was significantly lower than that in children exposed to 3000 K light. In children, blue‐enriched LED lighting has a greater impact on melatonin suppression and it inhibits the increase in sleepiness during night. Light with a low color temperature is recommended at night, particularly for children's sleep and circadian rhythm.

## Introduction

Melatonin is an endogenous hormone secreted from the pineal gland in the brain, and its secretion is known to be related to the regulation of circadian rhythm and the sleep/wake cycle (Sack et al. 2000; Cajochen et al. 2003; Arendt 2005). In humans, the suprachiasmatic nucleus (SCN), the circadian pacemaker, reduces melatonin production by the pineal gland during daylight hours and induces the release of melatonin at night. It is well known that nocturnal melatonin secretion is acutely suppressed not only by exposure to bright light but also by exposure to ordinary room lighting (Gooley et al. 2011) or even exposure to light with an illumination level of less than 100 lux (Zeitzer et al. 2000). Although a causal relationship between pineal melatonin and health risks has not been revealed yet, light‐induced melatonin suppression at night has been considered to be a possible factor causing several health risks in human (Navara and Nelson 2007; Kantermann and Roenneberg 2009; Stevens and Zhu 2015).

In ordinary daily routines, however, humans are exposed to artificial lights at home until bedtime. Children commonly sleep earlier than adults. This suggests that children might be less affected by evening light since the magnitude of melatonin suppression varies depending on the duration of light exposure (Aoki et al. 1998) as well as light intensity (Mcintyre et al. 1989). In contrast to this possibility, however, we have recently found that nocturnal melatonin suppression in children is greater than that in adults in response to identical light conditions (Higuchi et al. 2014): the percentage of melatonin suppression in children was approximately two‐times greater than that in adults. According to the estimation of age‐dependent circadian photosensitivity (Turner and Mainster 2008), circadian photoreception of 10‐year‐old children was twice than that of 45‐year‐old adults due to age‐related losses in crystalline lens transmittance and pupillary miosis. Although it is still controversial whether aging is related to retinal sensitivity, age‐related alterations in pupil size (Winn et al. 1994) and the crystalline lens (Boettner and Wolter 1962; Barker et al. 1991) have been suspected as factors involved in melatonin sensitivity (Charman 2003; Herljevic et al. 2005).

The short wavelength of visible light (i.e., blue light) is known to be the most powerful photic stimulus suppressing melatonin (Brainard et al. 2001; Thapan et al. 2001). Polychromatic white light, that is, blue‐enriched white light, has also been shown to have a great impact on human physiological functions (Deguchi and Sato 1992; Morita et al. 1995; Kozaki et al. 2008; Chellappa et al. 2013; Brainard et al. 2015). The results of those studies are closely related to the high photosensitivity of melatonin‐containing retinal ganglion cells (mRGCs) to blue light. It is now well established that the retinohypothalamic inputs of photic signals from mRGCs into various brain areas (Daneault et al. 2016) greatly contribute to non‐image forming responses such as circadian photoentrainment and light‐induced melatonin suppression. Coincidently, the age‐related increase in lens absorbance becomes remarkable particularly for the short wavelength range, suggesting that the difference between melatonin sensitivity in children and that in adults is more apparent under a high color temperature lighting condition than under a low color temperature lighting condition. Melatonin is also involved in the modulation of other physiological functions including sleepiness, body temperature, and autonomic function (Cajochen et al. 2005). The aim of the present study was therefore to determine the difference in effects of high‐color and low color temperatures of LED lighting on pineal melatonin production and subjective sleepiness in children and adults.

## Materials and Methods

### Participants

Twenty‐two healthy children (12 boys and 10 girls) and 20 of their parents (adults) (nine men and 11 women) participated in this study. All of the participants had no sleep complaints and were not taking any medications. The mean ages and standard deviations of the adult group and child group were 41.7 ± 4.4 years and 8.9 ± 2.2 years, respectively. Prior to the experiments, oral and paper‐based explanations for the children and their parents were conducted. Then the participants provided written informed consent, which was approved by the Ethical Committee of Kyushu University. Informed consent forms for the children were completed by their parents after confirming their child's agreement for participation. The study was registered at the University Hospital Medical Information Network (UMIN) Clinical trial (ID: UMIN000031391).

### Experimental conditions and procedures

A between‐subjects design with four combinations, including two age groups (adults and children) and two color temperature conditions (low and high), was adopted in this study. The participants were divided into four experimental groups, including adults with high color temperature (Adult‐H) or low color temperature (Adult‐L) and children with high color temperature (Child‐H) or low color temperature (Child‐L). There were no significant differences in age, sex distribution, habitual bedtime and wake time measured by a sleep diary for 10 days before the start of the experiment between the child groups (Child‐L vs. Child‐H) and between the adult groups (Adult‐L vs. Adult‐H) (Table 1).

**Table 1:** Characteristics of each group (sex ratio, age, and sleep habits)

Group	*n*	Sex (M:F)	Age (SD)	bed time (SD)	Wake time (SD)
Adult‐L	10	5 : 5	41.3 (4.5)	23:24 (1:26)	6:41 (0:56)
Adult‐H	10	4 : 6	42.0 (4.4)	23:33 (0:50)	6:25 (0:38)
Child‐L	10	7 : 3	8.9 (2.4)	21:53 (0:39)	6:52 (0:29)
Child‐H	11	5 : 6	8.9 (2.2)	21:42 (0:55)	7:02 (0:33)

**Figure 1:**
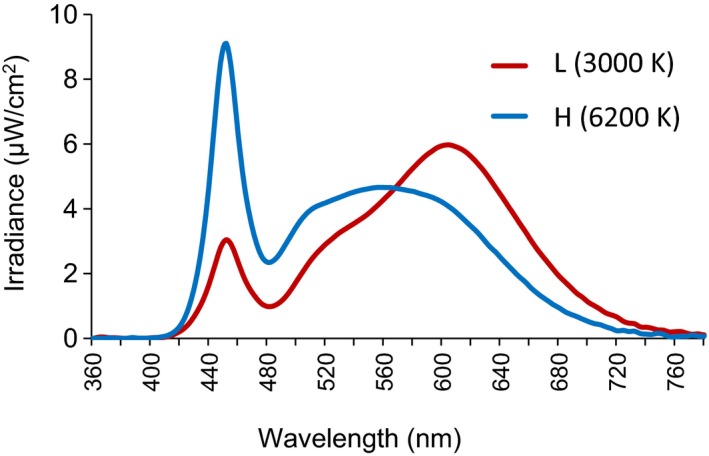
Spectral irradiance at eye level in light with a high color temperature and light with a low color temperature.

All experimental conditions were designed to be close to the lighting environment in actual life. A light‐emitting diode (LED) ceiling light (HH‐LC569A, Panasonic Inc., Japan), for which color temperature can be adjusted, was used for exposure of participants to light in the experiments. The color temperature conditions were 3000 K as a low condition and 6200 K as a high condition (Fig. 1). Each color temperature condition matched at around 14.4 log photons/cm^2^/sec. The illuminance levels of low‐ and high‐color temperature conditions were 299.0 and 305.2 lx in the vertical direction from the height of the subject's eyes, respectively. The amounts of melanopic illuminance were 149.2 at 3000 K and 292.9 at 62,000 K. Information on each condition is given in Table 2. An illuminance spectroradiometer (CL‐500A, KONICA MINOLTA INC., Japan) was used in the measurements. To minimize the difference between eye heights of children and adults during the light exposure session, we adjusted the height of chairs with consideration of their sitting height.

**Table 2:** Description of the light conditions

	Unit	Low	High
Color temp.	K	3041	6218
Melanopic lux (m‐lux)	–	149.2	292.9
Illuminance	lx	299.0	305.2
Photon density	log_10_ (1/cm^2^/sec)	14.44	14.43

The experiment was conducted for two consecutive nights in September and October at an accommodation facility in Japan. All participants were instructed to keep their habitual sleep schedule and to record a daily sleep diary for 1 week before the start of the experiment. The children usually went to bed at 21:41 ± 0:50 and woke up at 6:49 ± 0:28, whereas their parents went to bed at 23:18 ± 1:07 and woke up at 6:20 ± 0:48. Since the participants maintained their habitual sleep schedule prior to the experiment, the end time of the experiment (i.e., experimental bedtime) was determined on the basis of each participant's habitual bedtime. Unfortunately, the experiment had to be finished by 0:00 h according to the operation policy of the accommodation facility. Consequently, considering the daily variation of habitual bedtime, each participant's experimental bedtime was determined as one hour longer than the previously reported habitual bedtime: 22:00 (*n* = 4) and 0:00 (*n* = 6) in the Adult‐L group; 22:30 (*n* = 2), 23:00 (*n* = 1), and 0:00 (*n* = 7) in the Adult‐H group; 21:30 (*n* = 2), 22:00 (*n* = 3), 22:30 (*n* = 2), 23:00 (*n* = 3), and 0:00 (*n* = 1) in the Child‐L group; and 21:30 (*n* = 2), 22:00 (*n* = 1), 22:30 (*n* = 3), 23:00 (*n* = 3), and 0:00 (*n* = 2) in the Child‐H group. Average light exposure durations and standard deviations in the Adult‐L, Adult‐H, Child‐L and Child‐H groups were 4:40 ± 0:48, 4:34 ± 0:47, 3:24 ± 0:45 and 3:33 ± 0:45, respectively. The durations in children were significantly shorter than those in adults (Adult‐L vs. Child‐L: *P *=* *0.008; Adult‐H vs. Child‐H: *P *=* *0.016, unpaired two‐tailed Student's *t*‐test) but no significant difference was found between the adult groups (*P *=* *0.315) or between the child groups (*P *=* *0.514) (unpaired two‐tailed Student's *t*‐test). On the first night, participants spent time in a dimly lit room (<30 lx) from 19:00 until one hour after each habitual bedtime (BT+1 h). On the second day, the participants woke up at their respective habitual wake times. Participants were asked to avoid excessive physical activity and to wear a hat outside during the day. Both the adults and children participated in either the experimental condition of 3000 K or 6200 K from 19:00.

Saliva samples were collected at 1‐hour intervals from 19:00 (or 19:30 for some participants who finished the experiment at 21:30 or 22:30) until the end of the experiment using a plain cotton plug (Salivette Sarstedt, Germany) on the first night and the second night. Also, subjective sleepiness was measured with the Karolinska Sleepiness Scale (KSS) at BT and BT+1 h on each day. During the experiment, participants were allowed to talk with each other, read a book, and play board games.

### Sample analysis

Salivary melatonin concentrations were measured by a radioimmunoassay kit (RK‐DSM; Bühlmann Laboratories AG, Allschwil, Switzerland). In order to evaluate the overall suppressive effect of light color temperature on melatonin, relative percent change in melatonin area under the curve (AUC; trapezoidal approximation) was calculated on the basis of melatonin AUC under the dim condition. However, data for subjects who showed a very low melatonin level (e.g., still <3 pg/mL at BT). The time of dim light melatonin onset (DLMO) was determined by linear interpolation between two time points at which melatonin concentration crossed the 4.0 pg/mL threshold. The percent change in AUC (AUC %change) was calculated as follows:

$$\text{AUC}\;\%\text{change}=100\times \left({\text{AUC}}_{\dim}-{\text{AUC}}_{\mathrm{light}}\right)/\left({\text{AUC}}_{\dim}\right)$$

Hence, higher AUC %change indicates a greater suppressive effect of the light condition on the melatonin profile.

The assay has a sensitivity of 0.2 pg/mL (0.9 pmol/L) and intra‐ and interassay coefficients of variation were below 4.8% and below 8.4%, respectively.

### Data analysis

All results are presented as means ± standard error. SPSS 23.0 ((IBM^©^ SPSS^©^ Statistics) was used for statistical analysis. Comparison of AUC %changes was made using a two‐sided, independent‐sample Student's *t*‐test or Welch's *t*‐test separately between age groups (i.e., Adult‐H vs. Child‐H or Adult‐L vs. Child‐L) and between different color temperature groups (i.e., Adult‐H vs. Adult‐L or Child‐H vs. Child‐L). Subjective sleepiness (KSS) was analyzed separately in each age group using repeated‐measures two‐way analysis of variance with groups with 3000 K or with 6200 K as a between‐subject factor and measurement time (BT, BT+1 h) as an independent variable. *P *<* *0.05 was considered to be statistically significant.

## Results

Experimental data for four adults (two men and two women) in the Adult‐L group and for three adults (one man and two women) in the Adult‐H group were excluded from analysis due to very low melatonin concentrations (below 3 pg/mL at BT). Average times of DLMO and standard deviations in the Adult‐L, Adult‐H, Child‐L, and Child‐H groups were 21:30 ± 0:53, 21:51 ± 0:53, 20:33 ± 0:45, and 20:33 ± 0:50, respectively. The unpaired two‐tailed Student's t‐test showed no statistically significant difference between the adult groups or between the child groups. However, DLMO in children was significantly earlier than that in adults (Adult‐L vs. Child‐L: *P *=* *0.040; Adult‐H vs. Child‐H: *P *=* *0.007).

**Figure 2:**
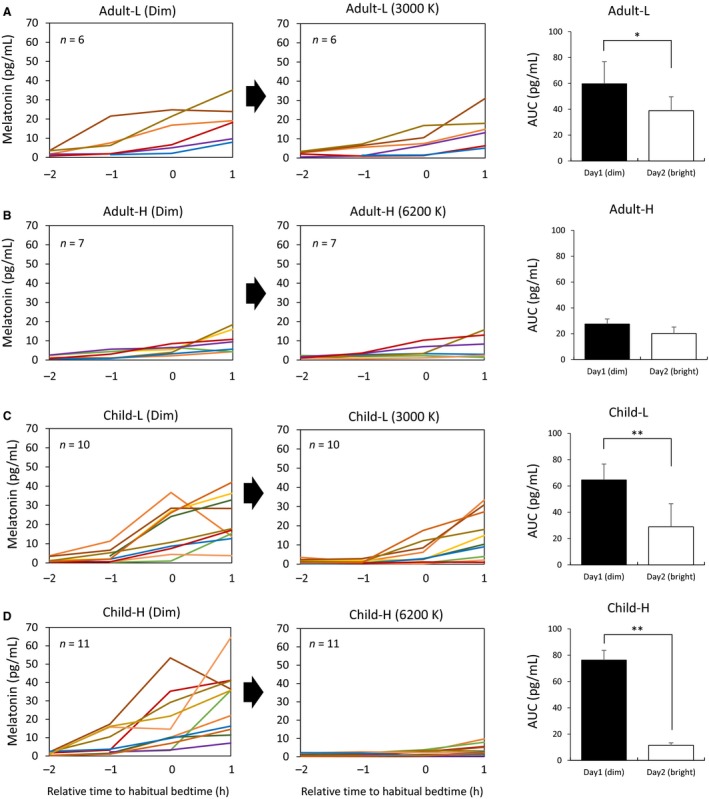
Salivary melatonin profiles (left and middle columns) and melatonin AUC (right column) in adults (A and B) and children (C and D). Individual melatonin data are shown (left and middle). 0 h means the habitual bedtime of each participant. **: *P *<* *0.01, *: *P *<* *0.05.

Figure 2 shows melatonin profiles of each group during the experimental conditions from 2 h before habitual bedtime to 1 h after habitual bedtime. Overall, the children in both groups secreted melatonin earlier and secreted more melatonin than did the adults. As the graphs for individual melatonin change show, melatonin secretion in children was clearly suppressed by the light exposure regardless of color temperature. Compared with melatonin under the condition of dim light, lights at both of 3000 K and 6200 K significantly reduced melatonin concentrations in children (*P *=* *0.001; *r *=* *0.83 and *P *=* *0.004; *r *=* *0.79, respectively). In adults, exposure to 3000 K light induced statistically significant melatonin suppression compared with melatonin concentrations under a dim condition (*P *=* *0.049; *r *=* *0.76). However, exposure to 6200 K light induced melatonin suppression at a weak significance level (*P *=* *0.091; *r *=* *0.64).

Table 3 shows light‐induced melatonin AUC %change relative to the AUC under the dim condition in each group. A comparison of the different color temperature groups showed that the AUC %change was significantly different between the child groups (Child‐L: 58.1%; Child‐H: 81.2%, *P *=* *0.012; *r *=* *0.55) but not between the adult groups (Adult‐L: 30.4%; Adult‐H: 30.4%, *P* = 0.999). A comparison between different age group showed that AUC %change was significantly different between the Child‐H group and the Adult‐H group (*P *=* *0.004; *r *=* *0.84) and between the Child‐L group and the Adult‐L group (*P *=* *0.047; *r *=* *0.50).

**Table 3:** Percentage of melatonin suppression (AUC)

Light condition	Adult	Child	
	Mean (%) (SE)	Mean (%) (SE)	
L (3000 K)	30.4 (10.9)	58.1 (7.4)	*P = *0.047*
H (6200 K)	30.4 (11.7)	81.2 (3.7)	*P = *0.004**
	*P = *0.999	*P = *0.012*	

**P < 0.01; *P < 0.05.

**Figure 3:**
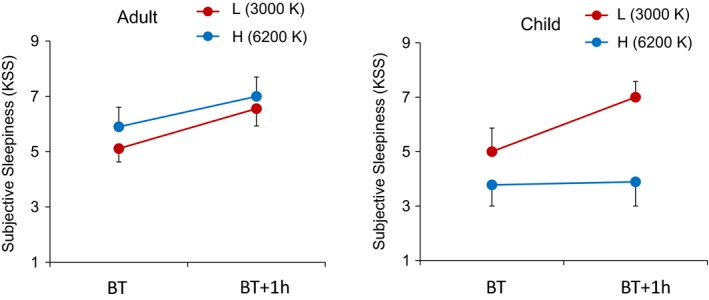
Subjective sleepiness (KSS) at habitual bedtime and one hour after habitual bed time.

In the analysis of subjective sleepiness (Fig. 3) in the adult groups, there were main effects in measurement time (*F*_1, 17_ = 17.902, *P* < 0.01) but no main effects in color temperature (3000 K and 6200 K), and no interaction between those two factors was found. In the child groups, however, there were main effects in both measurement time (*F*_1, 16_ = 4.527, *P* < 0.05) and color temperature (*F*_1, 16_ = 4.727, *P* < 0.05) and there was a tendency for a significant interaction between measurement time and color temperature (*F*_1, 16_ = 3.624, *P *=* *0.075). Subjective sleepiness in the Child‐H group was significantly lower than that in the Child‐L group. Furthermore, subjective sleepiness in the Child‐H group remained low even at one hour after bedtime, though that in the Child‐L group increased.

## Discussion

In this study, we compared the effects of exposure to two different color temperatures (3000 K and 6200 K) of LED lighting, which are used as room lights in Japan, on melatonin suppression and sleepiness in children and adults. Regardless of the color temperature, exposure to the light remarkably diminished children's melatonin secretion before bedtime. In the adult groups, however, the results showed a statistically significant melatonin suppression in the Adult‐L group but not as strong as that in the Child‐L group and very weak melatonin suppression in the Adult‐H group. These results provide further supportive evidence following the results of our previous study (Higuchi et al. 2014) showing that children are more likely than adults to be affected by evening light. Related to these results, the large melatonin concentration due to earlier melatonin onset in the child groups might be responsible for the greater melatonin suppression in this study. However, given that the light exposure duration was shorter in children than in adults, it is important to note that a short duration of exposure to evening light can strongly suppress melatonin in children.

Age disparity in melatonin suppression emerged differently depending on the color temperature of light: melatonin suppression in the Child‐H group was approximately 2.7‐times greater than that in the Adult‐H group, while that in the Child‐L group was approximately 1.9‐times greater than that in the Adult‐L group. This indicates the possibility that the contribution of mRGC to nocturnal melatonin suppression was promoted by high transmittance of the crystalline lens in children, particularly for a short wavelength light (Boettner and Wolter 1962; Barker et al. 1991). Nonetheless, in terms of the effects of age‐related ocular alteration on physiological functions, there is still a lack of agreement among previous studies (Herljevic et al. 2005; Daneault et al. 2012; Higuchi et al. 2014; Najjar et al. 2014; Gimenez et al. 2016). According to a recent report, pre‐ to mid‐pubertal children showed significantly greater melatonin suppression than that in children in the late to post‐pubertal group (Crowley et al. 2015) although there might be no large difference in lens transmittance or pupil size between the two groups (Barker et al. 1991; Winn et al. 1994), indicating factors other than ophthalmologic characteristics might be involved in the higher sensitivity to evening light in pre‐ to mid‐pubertal children. Hence, to draw a conclusion regarding the relationship between ocular alteration and light‐induced physiological responses, not only measurements of lens density and transmittance (Teikari et al. 2012; Najjar et al. 2016) but also detailed information on spectral components reaching the retina are needed in a future study.

Unexpectedly, we could not detect remarkable melatonin suppression in adults in high color temperature conditions. The amount of melatonin secretion until bedtime in the adult groups, especially in the Adult‐H group, was probably too small to elicit light‐induced melatonin suppression. It has been reported that the amount of melatonin secretion in humans decrease with advance of the puberty stage (Crowley et al. 2012). Additionally, some of the adult subjects finished the experiment at a very early time (22:00 or 23:00), and others who usually sleep around midnight had to finish the experiment by 0:00 h due to the operation policy of the accommodation facility. This might be a reason for the low melatonin concentrations in adult subjects. Thus, further examination with more adult subjects should be conducted to reach a conclusion regarding this issue.

Sleepiness in children differed depending on the color temperature of light, whereas there was no significant effect of the color temperature of light in adults. Sleepiness in children was significantly lower under the condition of a high color temperature of light than under the condition of a low color temperature of light. Furthermore, sleepiness under the condition of a high color temperature of light was not increased at bedtime. This result is consistent with the effect of a high color temperature of light on melatonin suppression in children. An alerting response to a short wavelength of light has been found as well as melatonin suppression in children, although the causal connection between endogenous melatonin and sleepiness remains controversial (Ruger et al. 2005; Cajochen 2007).

It has been well established that exposure to light at night can induce a phase delay of circadian rhythm (St Hilaire et al. 2012). Recently, one field study revealed that artificial light even at home before bedtime could cause a delay of the circadian phase (Burgess and Molina 2014). Another study showed that the use of a high color temperature of light at home is correlated with delay of the circadian phase in children as well as in adults (Higuchi et al. 2016). Studies on the effects of exposure to light from self‐illuminating devices, including computer monitors, tablets and e‐readers, have also been conducted in adults (Higuchi et al. 2003; Cajochen et al. 2011; Chang et al. 2015). Taken together, the results indicate the possibility that the light‐induced phase delay in children is greater than that in adults. Furthermore the use of self‐illuminating devices at night might be a factor that can elicit delayed bed time and short sleep time in children (Hale and Guan 2015).

There are several limitations in the present study. It has been shown that melatonin suppression and circadian rhythm in humans can be changed by a prior light exposure history (Hebert et al. 2002; Smith et al. 2004; Chang et al. 2011). Although we instructed participants to wear a hat when they go outside during the day, we did not strictly control the light exposure history of each subject during the experiment. For reference, the children and their parents acted together when they were either inside or outside the accommodation facility. Next, the experiment started at 19:00, but the durations of light exposure were different for children and adults. This could have affected the results for melatonin suppression. As mentioned above, however, we focused on the effects of evening light in actual life rather than that in a laboratory experiment. For reference, the average light exposure times calculated on the basis of DLMO and the standard deviations in the Adult‐L, Adult‐H, Child‐L, and Child‐H groups were 2:09 ± 0:42, 1:42 ± 0:39, 1:50 ± 0:42, and 1:59 ± 0:39, respectively, and there was no statistically significant difference in the duration among the groups (*F*_3, 29_ = 0.558, *P *=* *0.647, one‐way ANOVA).

Interestingly, the existence of a compensative mechanism in photic sensitivity of the retina against the yellowing crystalline lens has been suggested. Najjar et al. (2014) reported that there was no remarkable difference in melatonin suppression between young subjects and elderly subjects despite the different levels of lens transmittance in the two groups. Similarly, Gimenez et al. (2014) found an attenuated filtering effect of a soft orange contact lens on melatonin suppression when the period for wearing the lens was prolonged. If there is an adaptational mechanism in the retina depending on the feature of external photic stimuli, then it is necessary to investigate how the compensative mechanism affects the retina in children.

## Conclusions

The results of this study suggest that, although the duration of exposure to evening light in children is shorter than that in adults, the impact of light is greater in children than in adults. Light‐induced melatonin suppression in children is greater than that in adults under conditions of both high color and low color temperatures of light, blue‐enriched high color temperature of LED lighting has a greater impact on melatonin suppression in children and inhibits the increase in sleepiness during the night in children. In this regard, evening light with a low color temperature is recommendable for children's sleep and circadian rhythm. However, it should be noted that even lighting with a low color temperature could be a factor of melatonin suppression in children when it's illuminance is adjusted brightly.

## Conflict of Interest

No conflicts of interest, financial or otherwise, are declared by the authors.
